# Characterization of an *Aedes* ADP-Ribosylation Protein Domain and Role of Post-Translational Modification during Chikungunya Virus Infection

**DOI:** 10.3390/pathogens12050718

**Published:** 2023-05-16

**Authors:** Ramesh Kumar, Divya Mehta, Debasis Nayak, Sujatha Sunil

**Affiliations:** 1 Vector Borne Diseases Group, International Centre for Genetic Engineering and Biotechnology, New Delhi 110067, India; 2 Department of Biosciences and Biomedical Engineering, Indian Institute of Technology, Indore 453252, India

**Keywords:** poly ADP-ribosylation, *Aedes aegypti*, tankyrase, PARP, chikungunya virus (CHIKV)

## Abstract

Poly ADP-ribose polymerases (PARPs) catalyze ADP-ribosylation, a subclass of post-translational modification (PTM). Mono-ADP-ribose (MAR) moieties bind to target molecules such as proteins and nucleic acids, and are added as part of the process which also leads to formation of polymer chains of ADP-ribose. ADP-ribosylation is reversible; its removal is carried out by ribosyl hydrolases such as PARG (poly ADP-ribose glycohydrolase), TARG (terminal ADP-ribose protein glycohydrolase), macrodomain, etc. In this study, the catalytic domain of *Aedes aegypti* tankyrase was expressed in bacteria and purified. The tankyrase PARP catalytic domain was found to be enzymatically active, as demonstrated by an in vitro poly ADP-ribosylation (PARylation) experiment. Using in vitro ADP-ribosylation assay, we further demonstrate that the chikungunya virus (CHIKV) nsp3 (non-structural protein 3) macrodomain inhibits ADP-ribosylation in a time-dependent way. We have also demonstrated that transfection of the CHIKV nsP3 macrodomain increases the CHIKV viral titer in mosquito cells, suggesting that ADP-ribosylation may play a significant role in viral replication.

## 1. Introduction

ADP-ribosylation is a common modification that occurs in all life domains, including prokaryotic and eukaryotic organisms. It entails the attachment of mono or polymer units of ADP-ribose to target molecules, such as DNA, proteins, and RNA [1,2,3], and is known to play a role in a number of biological functions, including DNA damage repair, telomere maintenance, stress response, immunological response, cell signaling, and cell proliferation [4,5,6]. Three sets of proteins called writers, readers, and erasers control the process of ADP (Adenosine diphosphate)-ribosylation. The writers convert nicotinamide adenine dinucleotide (NAD^+^) to nicotinamide (NAM) and ADP-ribose, then the latter attaches to target molecules. ADP-ribosyl transferases (ARTs) are commonly used for mono PARPs or Poly ADP-ribose polymerase (PARP), depending on whether they add only a single ADP-ribose (MARP) or multiple ADP-ribose units to target molecules [1,7]. Reader proteins, which contain one of the following domains such as macrodomain, WWE domain (named after three conserved single letter amino acid residues), PAR-binding motifs (PBMs), or PAR-binding zinc finger (PBZ) domain, are able to recognize ADP-ribosylation on target proteins [7]. These proteins have important roles in localization [8], DNA damage response [9], and ubiquitin-mediated proteasomal degradation [10,11] by interacting with ADP-ribosylated proteins. Eraser proteins bind to and remove ADP-ribosylation. They are divided into: terminal ADP-ribose protein glycohydrolase 1 (TARG1), poly ADP-ribose glycohydrolase (PARG), and ADP-ribosyl-acceptor hydrolases (ARH1 and ARH3). Macro D1 and macro D2, Macro Ds, ARH1, ARH3, and TARG all eliminate mono-ADP-ribose residues, but PARG eliminates poly ADP-ribose chain [7,12].

In addition to glutamate and aspartate, additional acidic amino acid residues such as serine, arginine, and cysteine also act as acceptors for ADP-ribosylation by PARPs [2]. The next step is polymer extension, which entails repeatedly conjugating ADP-ribose from NAD+ to the previous ADP-ribose unit. This results in the construction of a linear ribose polymer chain (1″2′) made up of 2–200 ADP-ribose units, also known as a poly ADP-ribose (PAR) chain. Furthermore, branching is added to the PAR chains to boost both complexity and biological responsiveness [13,14,15]. By causing phase separation and encouraging protein–protein interactions, the PAR chains also aid the development of protein complexes [16].

The ADP-ribosylation process is also involved in the antiviral immune response [17,18,19], where it is known to activate different components in the immune pathway, such as ion channels [20], modulation of expression of genes involved in inflammation [21,22,23], and the RNAi (RNA interference) pathway [6], ultimately triggering the host defense mechanism against virus infection. It is well known that viruses of the *Coronaviridae*, *Togaviridae* and *Hepeviridae* families contain macrodomains, which hinder host-mediated immune response [24] by targeting stress granule formation [25], inhibiting the release of pro-inflammatory cytokines and interferons [26], enhancing pathogenesis [27], and promoting viral proliferation [28,29].

*Aedes aegypti* (*Ae. aegypti*) is an important vector for arboviruses, including dengue, Zika, chikungunya, yellow fever [30,31]. The effects of ADP-ribosylation during arboviral infections in the vector and function of the proteins implicated in the alteration in their survival are poorly understood. The non-structural protein 3 (nsP3) of the Chikungunya virus (CHIKV), an alphavirus, encodes a macrodomain that possesses ADP-ribosylhydrolase activity, which is important for virus replication and virulence [28,32]. In this study, we cloned and expressed the catalytic domain of *Ae. aegypti* tankyrase protein. The in vitro assay using the purified catalytic domain showed that the domain was able to auto-PARylate. When attached as monomer or polymer to target proteins, ADP-ribose moieties are known to alter their activity as well as have role in protein complex formation. The macrodomain is known to hydrolyze the ADP-ribose from the proteins and favor viral growth [25,33,34]. We found that PARylation was inhibited by the nsP3 protein, which is known to have a macrodomain, and its transfection into mosquito cells favored the CHIKV replication, indicating the crucial role of ADP-ribosylation and macrodomain play in deciding the outcome of mosquito-virus interaction.

## 2. Materials and Methods

### 2.1. Sequence Alignment and Phylogenetic Analysis

To identify PARP orthologs in *Ae. aegypti*, 17 human PARPs were blast aligned against known *Ae. aegypti* PARPs. Sequences were aligned and a phylogenetic tree was generated using MEGA 11 software [35]. The sequence alignment was done with MUSCLE algorithm. The sequences were then analyzed for phylogenetic analysis using the following method: statistical method: maximum likelihood, test of phylogeny: bootstrap method, no. of bootstrap replication: 1000, substitution model: Poisson model, ML heuristic method: nearest-neighbor-interchange (NNI), no. of threads: 5. Next, the *Aedes* PARP sequences were analyzed for domains present in them using ScanProsite (https://prosite.expasy.org/scanprosite/; accessed on 16 April 2022).

### 2.2. Cells, Virus, Infection and Transfection

Vero cells (ATCC^®^ CCL-81™) were purchased from ATCC and *Ae. albopictus* (C6/36) cells were obtained from NCCS, Pune, India. *Ae. aegypti*-derived cells (Aag2) were a kind gift from Dr. Kevin Maringer, The Pirbright Institute, Surrey, UK. C6/36 and Vero cells were maintained in DMEM medium as mentioned previously [36]. Aag2 cells were grown in Leibovitz’s L-15 Medium (Thermo Scientific Inc., Waltham, MA, USA, Cat. no. 11415064) and supplemented with 20% fetal bovine serum (Thermo Scientific Inc., Waltham, MA, USA, Cat. no. 10438018).

A lab-adapted CHIKV clinical strain, IND-2010#01 (Accession no. JF950631.1) was used to infect Aag2 cells [37], as mentioned in a previous study [36]. Vero cells were grown to full confluency and in Aag2 cells, 1 × 10^6^ cells/well in a 12-well plate were seeded. The next day, the medium in the wells was changed with serum-supplemented medium absent of antibiotics. EGFP and macrodomain cloned pIB/V5-His plasmids (1.5 µg each) were suspended in 100 µL serum-free medium with 2 µL TransIT transfection reagent (Mirus Bio LLC, Madison, WI, USA) separately and kept at RT for 20 min (minutes). The mixture was added drop-wise to cells and after 24 h (hour) the cells were infected with CHIKV at MOI (multiplicity of infection) of 1. The cells/supernatant were collected at 24, 36, and 48 hpi (hours post infection).

### 2.3. Gene Cloning, and Expression

To clone the catalytic domain tankyrase of *Ae. aegypti*, the protocol uses RNA isolated from *Ae. aegypti* and PrimeScript One Step RT-PCR Kit (Takara Bio Inc., Japan, Cat RR055B) tankyrase-specific primers: forward primer 5′-ATAGGTACCAGCGGCACATCCATGGCCAACAG-3′ and reverse primer 5′-ATAGGATCCCTCGCTGGCTCCTGGGGGCTAG-3′, with annealing at 65 °C and primer extension for 120 s (seconds). The PCR product was cloned into pET32a vector. The tankyrase-pET32a plasmid was transformed into *E. coli* CodonPlus cells, whereas CHIKV nsP3 cloned in pET29a was used from a previous study [38]. EGFP was also cloned using forward primer 5′-GGGGTACCATGCATCATCACCATCACCATCGGATGGTGAGCAAGGGCGAG-3′ and reverse primer 5′-ACCGGATCCCTTGTACAGCTCGTCCATGCCGAGAGTGATCCCG-3′, and CHIKV nsP3 macrodomain was cloned using forward primer 5′-ATAGGTACCATGGCACCGTCGTACCGGGTAAAACG-3′ and reverse primer 5′-ATAGGATCCGTCCGCATCTGTATGGCCTCAG-3′ into the pIB/V5-His vector.

### 2.4. Protein Purification

The tankyrase catalytic domain and CHIKV nsP3 protein were purified using a previously published protocol with slight modifications [38]. *E. coli* cultures having plasmid encoding *Ae. aegypti* tankyrase catalytic domain and CHIKV nsP3 were induced with 1mM IPTG for 16–20 h at 18 °C (degree Celsius). The cultures were pelleted and lysed in the lysis buffer (Tris-Cl (pH 8.0) 50 mM (milli Molar), NaCl 150 mM, EDTA 2 mM, glycerol 5%, β-mercaptoethanol 2 mM, and PMSF 1 mM) with lysozyme. This was followed by centrifugation. The clarified supernatant was mixed with freshly recharged Ni-NTA (Nickel-Nitrilotriacetic acid) agarose beads. The beads were eluted with lysis buffer containing 300 mM imidazole. The imidazole was removed by using dialysis membrane overnight with Tris-Cl (50 mM) pH 8.0, NaCl (150 mM), and DTT (2 mM) for further use of protein.

### 2.5. SDS-PAGE, Transfer, and Western Blotting

The SDS-PAGE and western blot were carried out according to prior methodology [36]. Briefly, Aag2 cells were lysed in RIPA buffer and 15–20 µg of each cell lysate or 4–6 µg of purified protein samples were resolved in SDS-PAGE gel and then transferred onto the nitrocellulose membrane (Bio-Rad Laboratories, Hercules, CA, USA). The membranes were probed with the following primary antibodies: anti-His HRP antibody (Santa Cruz, Dallas, TX, USA, Cat. no. sc-8036-HRP, 1:5000), anti-actin HRP (C4) (Santa Cruz, Dallas, USA, Cat. no. sc-47778 HRP, 1:6000), anti-pADPr antibody (10H) (Santa Cruz, Dallas, USA, Cat. no. sc-56198, 1:3000 dilution), and anti-V5 tag antibody (Thermo Fisher Scientific, Waltham, MA, USA, Cat. no. R960-25, 1:5000) and anti-CHIKV E1 (in house raised in mice, 1:3000). The blots probed with anti-pADPr antibody, anti-CHIKV E1 sera, and anti-V5 tag antibody were incubated with anti-mice IgG HRP antibody (Novus Biologicals, Colorado, USA, Cat. no. NB7539, 1:6000 dilution), washed with PBST, and then visualized using the Bio-Rad ChemiDoc MP System (Bio-Rad Laboratories, Hercules, CA, USA) after brief exposure to a chemiluminescent substrate. The uncropped images are included in supplementary information Figure S1.

### 2.6. Plaque Assay

The viral titration was done using plaque assay as per previously published protocol [39]. Briefly, the medium was replaced with serum-free medium 1 h prior to infection. The medium collected from CHIKV-infected Aag2 cells were initially diluted at 1:10 and added to the first well in triplicates, and then was diluted at 1:2 in the rest of the wells. The virus was allowed to bind to the cells for 90 min, and then the serum-supplemented medium was added. The wells were then added with 1% carboxymethyl cellulose (CMC) (Sigma–Aldrich, St. Louis, MO, USA, Cat. no. C4888) and plates were transferred back to the 5% CO_2_ supplemented humidified incubator at 37 °C for 72 h. The cells were fixed with paraformaldehyde for 1 h. After fixing, crystal violet stain (0.25%) was added to the wells and incubated for 30 min. The stain solution was discarded and wells were rinsed with tap water. The plaques were calculated as plaque-forming units (pfu) = (number of plaques)/(dilution × volume of the virus).

### 2.7. In Vitro PARylation Assay and Co-Incubation Assay with CHIKV nsP3 Protein

The in vitro assay was performed following previous protocol [40]. The *E. coli* purified recombinant tankyrase protein (8 to 12 µg) was incubated for the specified time at 28 °C in PARP reaction buffer (50 mM Tris-Cl [pH 8.0], 4 mM MgCl_2_, 0.2 mM DTT (dithiothreitol) containing 25 µM beta-Nicotinamide adenine dinucleotide sodium salt (NAD+) (Sigma–Aldrich, St. Louis, MO, USA, Cat. no. N0632-1G). The reactions were terminated by adding SDS loading buffer, and 4–6 µg of each protein sample was fractionated by 10% SDS-PAGE. The proteins were transferred onto a nitrocellulose membrane and probed with anti-pADPr antibody (10H) (Santa Cruz, USA, Cat. no. sc-56198 1:2000 dilution). The membrane was then probed with anti-mice IgG HRP antibody (Novus Biologicals, Centennial, CO, USA, Cat. no. NB7539) and visualized in a Bio-Rad ChemiDoc MP System after brief exposure to chemiluminescent substrate. Similar to the in vitro PARylation assay, the nsP3 co-incubation assay was carried out. The purified recombinant CHIKV nsP3 protein (8 to 12 µg) was added to the reaction mixture for the desired time and the reaction was stopped by adding SDS loading buffer. The 4–6 µg of each sample were then separated by SDS-PAGE, transferred onto the nitrocellulose membrane, and probed with anti-pADPr antibodies, followed by exposure to chemiluminescent substrate and visualization in ChemiDoc MP system.

### 2.8. In Vitro Transcription, RNA Isolation and Real-Time PCR

For double stranded RNA synthesis, T7 sequence (Tankyrase forward primer 5′-TAATACGACTCACTATAGGGTCACCGAACTGCTCATCAAG-3′, reverse primer 5′-TATCCGAAGCGAAAGCAAGTCCCTATAGTGAGTCGTATTA-3′, EGFP forward primer 5′-TAATACGACTCACTATAGGGATGGTGAGCAAGGGCGAGG-3′, and reverse primer 5′-TAATACGACTCACTATAGGGCTTGTACAGCTCGTCCATGCC-3′) was added to the primer and the desired product of around 400 bp was amplified using Dream Taq DNA polymerase (Thermo Fisher Scientific Inc., Waltham, MA, USA, Cat. no. EP0701) following the manufacturer’s protocol. The PCR product was purified and an in vitro reaction was setup using MEGAscript T7 transcription kit (Thermo Fisher Scientific Inc., Waltham, MA, USA, Cat. no. AM1334) following the manufacturer’s protocol. The dsRNA after in vitro transcription was treated with TURBO DNase. The RNA was purified using TRIzol reagent. Whole cell RNA isolation from Aag2 cells was done following previous protocol [41]. Total cellular RNA was isolated using TRIzol (Thermo Fisher Scientific Inc., Waltham, MA, USA). RNA was dissolved in DEPC treated water and quantified. One-step SYBR green real-time PCR was carried out on PIKOREAL 96 Well real-time PCR system (Thermo Fisher Scientific Inc., Waltham, MA, USA). A total of 300 ng total RNA per reaction was used with 0.3 μM of each primer with QuantiTect PCR kit (Qiagen, Hilden, Germany). The RT-PCR conditions for the one-step RT-PCR consisted of a 30 min reverse transcription step at 50 °C and then 2 min of initial denaturation at 95 °C, followed by 40 cycles of PCR at 95 °C without holding time (denaturation), 60 °C for 30 s (annealing), and 72 °C for 30 s (extension). Small subunit ribosomal protein 7 (RPS7) was used as an internal control. Tankyrase real-time PCR sequence used forward primer 5′-GGTGAAGAACCTCGAGAAAGAA-3′ and reverse primer 5′-CAATAGCAGCAAAGCTGGAAC-3′ and RPS7 forward primer 5′-CCCGGTTGACGATGGATTT-3′ and reverse primer 5′-TCACGAAACCAGCGATCTTATT-3′.

### 2.9. Immunofluorescence Assay

Aag2 cells were cultured in 6 well plates containing sterile glass coverslips. Cells were infected with CHIKV at MOI 1 for 24 h and 48 h. The cells were then fixed with 4% paraformaldehyde for 30 min and then permeabilized using 0.1% Triton-X-100 for 30 min. Cells were then blocked with bovine serum and then incubated with anti-CHIKV nsP3 rabbit serum [38] at 1:200 dilution in PBS (phosphate buffer saline) + 2.5% BSA overnight. The following day, washing was done using PBST (PBS + 0.1% tween-20) 3 × 10 min. The cells were then added with a secondary antibody (anti-mice IgG Alexa 594) at 1:400 dilution. This was followed by washing with PBS + 0.1% tween-20 for 3 × 10 min. The cells were immersed in DAPI for a few minutes and then visualized in Nikon eclipse confocal microscope (Nikon Corp, Tokyo, Japan) with oil immersion for magnification.

### 2.10. Statistical Analysis and Software

Statistical analyses for plaque assay analysis and real-time PCR were performed using two-way ANOVA. The analyses were done using Graphpad prism software (version 9.1.1).

## 3. Results

### 3.1. Identification of ADP-Ribose Polymerases in Ae. aegypti

**Figure 1:**
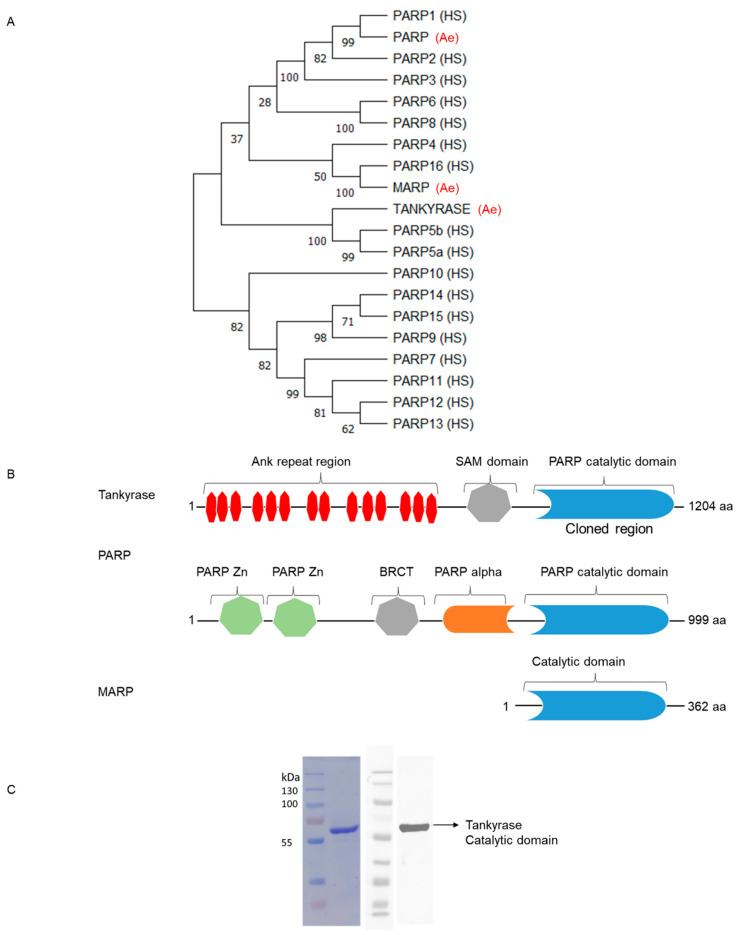
Analysis of ADP-ribose polymerase proteins in *Ae. aegypti*. (**A**) Phylogenetic comparison of 17 human PARP proteins with those from *Ae. aegypti*. *Aedes* PARPs are highlighted by red color. HS = *Homo sapiens*, Ae = *Ae. aegypti*; (**B**) Domain analysis of the ADP-ribose polymerase proteins from *Ae. aegypti*. Each color denotes a certain sort of domain, and the quantity of colored boxes indicates how many copies of that particular domain in the protein, and (**C**) Coomassie staining and western blot of purified recombinant tankyrase catalytic domain and western blot with anti-His HRP tagged antibody.

In order to compare the proteins in *Ae. aegypti* to the 17 human PARPs, sequence alignment was performed using blast tool (Blastp, NCBI). The results showed that *Ae. aegypti* encodes for three ADP-ribose polymerases, which are as follows: (1) tankyrase (NCBI accession: XP_021708496.1), (2) Poly ADP-ribose polymerase (PARP; NCBI accession: XP _001661932.1), and (3) Mono-ADP-ribose polymerase (MARP; NCBI accession: XP _001647568.1). The phylogenetic sequence analysis showed that the *Ae. aegypti* tankyrase protein was showing the highest sequence similarity to human PARP5a and PARP5b, and the *Ae. aegypti* PARP protein had the highest sequence similarity to human PARP1, while the *Ae. aegypti* MARP protein was showing the highest sequence similarity to human PARP16 (Figure 1A). These three ADP-ribose polymerases differed from one another in terms of the various sorts of domains. Here is the domain analysis for the three proteins:

1. **Tankyrase:** Tankyrase-1 (PARP5a) and tankyrase-2 (PARP5b) in human were found to be closest to *Ae.* tankyrase among the 17 human PARPs (Figure 1A). Three different types of domains were identified by the domain analysis: Ankyrin repeats, SAM domains, and PARP catalytic domains (Figure 1B). The 30–35 amino acid long motifs known as ankyrin repeats, which have a helix-turn-helix shape, are essential for protein–protein interactions [41,42]. Protein–protein interactions are mediated by another domain called the sterile alpha motif (SAM). These play a role in oligomerization as well as binding [43]. ADP-ribose is added by the third domain, called the PARP catalytic domain. Sequence alignment of tankyrase and PARP5b revealed an Ankyrin repeat region, which is crucial for protein–protein interaction and PARP catalytic domain, which is responsible for ADP-ribosylation activity, exhibited a higher region of similarity (Figure S2).
2. **PARP:** A poly ADP-ribose polymerase called PARP is the other protein found in *Ae. aegypti*. The most resemblance between *Ae. aegypti* PARP and human PARP1 was found during the phylogenetic analysis (Figure 1A). According to domain analysis, there are different types of domains: PARP Zn, BRCT, PARP alpha, and PARP catalytic domain (Figure 1B). A zinc finger domain, PARP Zn, included two copies. These proteins, which typically reside in the nucleus, are implicated in DNA repair [44]. The BCRT (BRCA1 C-terminus) domain was the second domain from the protein’s N-terminal. When the PARP alpha domain binds to the site of DNA damage, it transmits the activation signal [45]. The sequence alignment of PARP with PARP1 revealed several amino acid similarities between these two proteins, with the PARP catalytic domain showing the highest degree of similarity, indicating that this domain is mostly conserved in these animals (Figure S3). All of these facts suggest that the *Aedes* PARP protein is an enzyme that repairs DNA damage.
3. **MARP:** Mono-ADP-ribose polymerase (MARP) is responsible for adding mono-ADP-ribose units to proteins. These proteins cannot further connect ADP-ribose subunits to the terminals of those already attached [46]. According to the results of the phylogenetic research, the human MARP protein PARP16 and the *Ae. aegypti* MARP have the highest degree of similarity (Figure 1A). Proteins share comparable amino acids in the region responsible for catalytic activity of the protein, as seen by the sequence alignment of MARP and PARP16 (Figure S4). The MARP protein from *Ae. aegypti* is 362 amino acids long and only comprises a catalytic domain (Figure 1B), suggesting that it may be used for priming proteins or for MARylating proteins that are either activated or inactivated upon MARylation.

Based on analysis and evidence from the literature, we came to the conclusion that tankyrase is responsible for attaching the ADP-ribose chain to proteins, PARP is responsible for DNA repair, and MARP is responsible for intracellular signaling or priming. As the *Ae. aegypti* tankyrase protein contained a region implicated in protein–protein interaction, we moved forward with its cloning, production, purification, and characterization. These kinds of proteins are crucial for controlling cellular functions, and their discovery and characterization may shed light on the intricate mechanisms governing numerous biological processes. Full-length PARP proteins are required for the PARylation of target proteins in cells [47,48,49], but the catalytic domain alone is sufficient to create ADP-ribose chains on the proteins [49]. The catalytic domain of *Ae. aegypti* tankyrase protein (Figure 1B) was cloned into pET32a vector. The purified protein (of 60 kDa (kilo Dalton) size) was expressed in soluble form and was checked for purity using Coomassie stain and western blot (Figure 1C).

### 3.2. In Vitro PARylation Assay of Catalytic Domain of Tankyrase Protein and Impact of nsP3 Macrodomain

**Figure 2:**
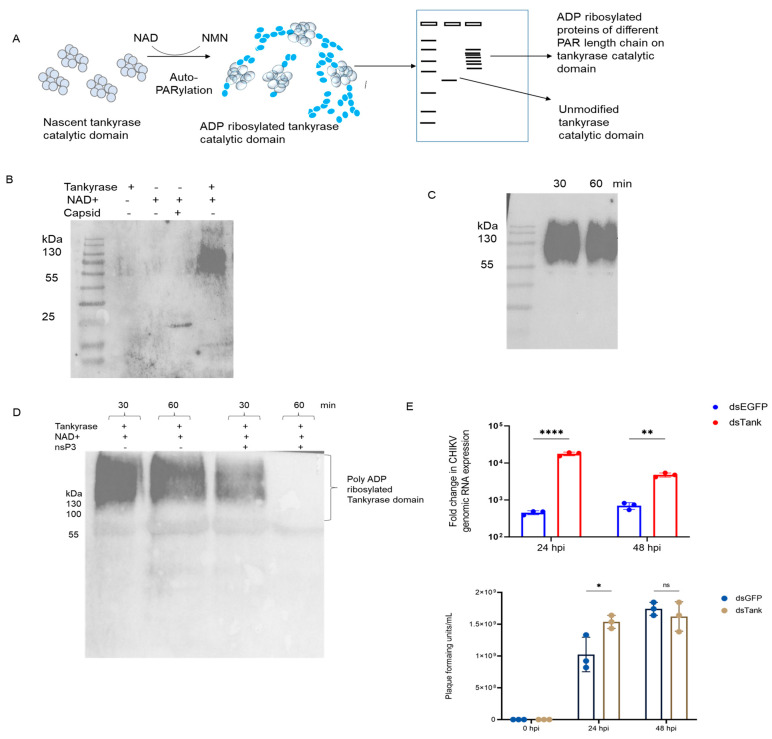
In vitro PARylation assay of tankyrase catalytic domain. (**A**) Schematic diagram of in vitro PARylation assay and sample (unmodified protein and ADP-ribose modified) protein separation on SDS-PAGE; (**B**) In vitro PARylation assay with tankyrase alone (negative control), NAD^+^ alone (negative control), CHIKV capsid proteins with NAD^+^ and tankyrase with NAD^+^ for 30 min reaction (left image is the anti-PAR antibody blotted membrane and right image is the Ponceau-stained membrane before blocking and anti-PAR antibody exposure); (**C**) Western blot of time points (30 and 60 min) of in vitro PARylated tankyrase catalytic domain; (**D**) In vitro PARylation assay in the absence of CHIKV nsP3 protein for 30 and 60 min (lane 1 and lane 2 from the left side). The impact of nsP3 protein on PARylation was checked by co-incubation of nsP3 protein and PARylation buffer having tankyrase protein for 30 and 60 min (lane 3 and 4) and (**E**) dsRNA mediated knockdown of *Ae. aegypti* tankyrase transcript. Aag2 cells were transfected with dsRNA for tankyrase and EGFP (control) for 24 h and then infected with CHIKV at MOI of 1. Cells were collected at 24 hpi and 48 hpi and viral titer was quantified by CHIKV E1 specific primers using real-time PCR and plaque assay. ns- non-significant, * *p*-value < 0.05 ** *p*-value < 0.001 and **** *p*-value < 0.0001.

**Figure 3:**
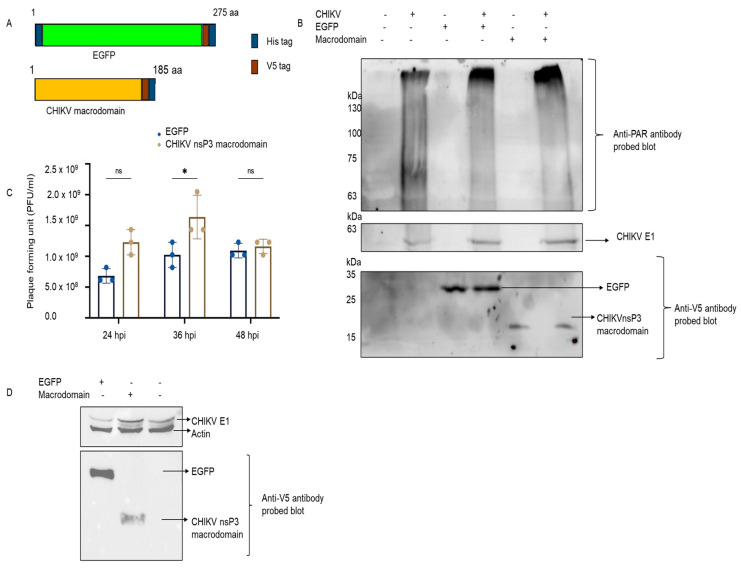
Effect of CHIKV nsP3 on PARylation. (**A**) Diagram of EGFP and CHIKV macrodomain cloned in insect vector (pIB/V5-His); (**B**) EGFP and CHIKV nsP3 macrodomain-pIB clones were transfected into Aag2 cells and then infected with CHIKV at MOI of 1 for 48 hpi. The lysates were blotted with mice anti-PAR antibody (for global PARylation level detection) and V5 (for detection of transfected EGFP and macrodomain in cells) and CHIKV E1 sera for different time points to observe the impact on viral growth (top), Ponceau-stained membrane sued to probing the antibodies (bottom); (**C**) Aag2 transfected with EGFP and macrodomain were infected with CHIKV at MOI of 1 for 24, 36, and 48 hpi. The medium was collected and used for viral titration via plaque assay, error bars represent standard deviation (sd). *n* = 4 (triplicates), and (**D**) Western blot of EGFP and macrodomain transfected cells infected with CHIKV at MOI of 1 after 48 hpi. The membrane was blotted with antibodies for CHIKV E1, actin, and V5 tag. ns- non-significant, * *p*-value < 0.05.

Each of the several domains that make up the PARP proteins is essential for their proper function in cells. Target proteins are added with long, variable-length ADP-ribose chains by these PARPs (Figure 2A) [15,16,50]. In this study, the capacity to add ADP-ribose subunits was initially assessed in the catalytic domain of tankyrase proteins. The tankyrase alone (Figure 3B, lane 1) and NAD^+^ (Figure 2B, lane 2) as well as CHIKV capsid protein (Figure 2B, lane 3) were employed as a negative control (for a non-specific signal). The presence of tankyrase catalytic domain resulted in an intense band of higher molecular-weight proteins (Figure 2B, lane 4), indicating that tankyrase domain was using NAD^+^ as a substrate to add ADP-ribose to itself via mechanism called auto-PARylation (Figure 3A). At both 30 min and 60 min, the PARylation assay revealed that the protein had undergone ADP-ribose modification with various lengths of the PAR chain (Figure 2C).

The in vitro PARylation assay showed that catalytic domain of tankyrase could add ADP-ribose units. Previous work from lab by Mathur et al. [51] has shown that CHIKV nsP3 macrodomain act as a viral suppressor of RNAi. CHIKV macrodomain is a mono-ADP-ribosylhydrolase and is crucial for the viral replication [28,52]. We were curious to find out if the CHIKV macrodomain, which is known to remove mono-ADP-ribose moieties [52], affected the poly ADP-ribosylation of proteins caused by *Ae. aegypti* PARPs. The bacterially purified CHIKV nsP3 protein (Figure S5) was added to the PARylation reaction mixture, and the reaction was run for 30 and 60 min in order to assess the function of the nsP3 macrodomain on ADP-ribosylation. Following the incubation of the nsP3 protein, it was found that the PARylation decreased as the incubation duration increased up to 60 min. In comparison to the 30 min and 60 min of the PARylated samples alone, the number or length of the PAR chains was lower at 30 min following the incubation of the nsP3 protein and dramatically decreased at 60 min (Figure 2D). The knockdown of tankyrase gene by dsRNA transfection resulted in increased titer of CHIKV (Figure 2E).

### 3.3. Effect of CHIKV nsP3 Macrodomain on PARylation Activity of Tankyrase

In CHIKV-infected Aag2 cells, the effect of the macrodomain alone on viral replication was also investigated. The nsP3 protein form discrete granules in cells, called replication complexes, and the number of cells increases with infection time (Figure S6), indicating that the nsP3 protein is not uniformly present in cells. To evaluate the impact of the macrodomain in viral kinetics, EGFP and the CHIKV macrodomain were cloned in the pIB/V5-His vector (Figure 3A) and transfected into Aag2 cells. The lysates were separated on SDS-PAGE gel and incubated with an anti-pADPr antibody. In all conditions of CHIKV infection (alone, with EGFP, or macrodomain transfected cells), PAR levels were increased compared to uninfected cells and transfected cells (Figure 3B). The global cellular PAR level difference between EGFP and macrodomain transfected cells was not significant. In plaque assay, we observed that the viral titer was high in macrodomain transfected cells compared to control (EGFP transfected) at 24 and 36 hpi, but at 48 hpi the difference between control and macrodomain was small (Figure 3C). At 24 hpi, western blot examination of CHIKV E1 protein revealed a similar pattern. CHIKV expression was lower in cells transfected with EGFP (lane 1) than it was in cells transfected with macrodomain (lane 2) (Figure 3D).

## 4. Discussion

ADP-ribosylation is an important PTM of proteins and nucleic acids that is mediated by PARPs. DNA repair, cell signaling, stress response, pathogen response, and gene control are just a few of the functions that PARPs are engaged in [49]. By targeting cellular transcripts, encouraging apoptosis [53], attenuating RISC (RNA induced silencing complex) mediated transcript silencing [54], inducing interferon-stimulated genes (ISGs), and degrading viral proteases, PARPs provide antiviral functions during viral infections [55]. In the current study, the sequence alignment with human PARPs led to the identification of three *Ae. aegypti* PARP proteins, including tankyrase (PARP5b), PARP (PARP1), and MARP (PARP16). Among these, tankyrase catalytic domain was cloned, expressed, and purified in a bacterial system. By using an in vitro PARylation assay, it was discovered that the tankyrase catalytic domain add PAR chains of variable length to its own molecules (auto-PARylation). The phosphate residues in the PAR chains imparts a negative charge, which interacts with the candidate proteins’ PAR binding motif (PBM) [56]. The length and degree of branching impacts the propensity to create multimeric complexes [50,57] and also affects cellular systems [15]. Knockdown of tankyrase led to higher viral titer, indicating that there might be other PARP isoforms that are involved in the immunity against viruses, or tankyrase is involved in the inactivation of viral proteins, hence its knockdown increasing the viral titer. A recent study highlights that mono-ADP-ribosylation of viral protein (nsP2) by host PARP leads to inhibition of nsP2 enzymatic activity [34], raising the possibility that a similar protein might be playing a role in providing immunity to mosquito cells against viral infection.

**Figure 4:**
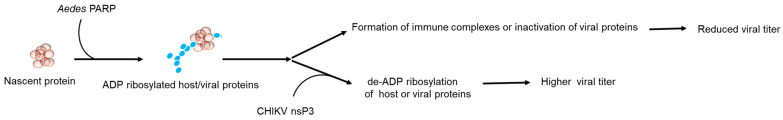
Proposed model of mechanism of tankyrase-mediated PARylation and CHIKV nsP3 macrodomain-mediated de-ADP-ribosylation of host/viral proteins. We hypothesize based on literature as well as current evidence that ADP-ribosylation inhibits viral growth by activating immune pathways or inactivating crucial viral proteins and thus reduces viral titer. CHIKV nsP3 has strong de-MARylation and weak de-PARylation activity. This indicates that nsp3 macrodomain either removes ADP-ribose from viral proteins, thereby preventing their inactivation or from host proteins thus inhibiting their role in immune pathways and crucial metabolic processes. This eventually leads to increased viral titer and compromised host immune system.

The fact that the active macrodomain of an alphavirus is conserved in the active site region shows how crucial the active macrodomain is for viral life [32]. According to earlier research, mutation in the active areas influences viral replication [28,32]. The results of the current study demonstrated that CHIKV infection leads to increased PARylation of the cellular proteome, but nsP3 macrodomain transfection did not affect the global PARylation compared to the EGFP control. During infection, nsP3 protein is present as discrete granules in the cells, indicating that it may not be interacting with the whole host proteome but instead only a limited number of proteins. Transfection of nsP3 macrodomain significantly reduced viral titer, suggesting that the macrodomain prevents PAR-chain formation by hydrolyzing ADP-ribose. Based on our data, we proposed a model hypothesis that host PARP proteins are either ADP-ribosylate host or viral proteins which leads to the inhibition of viral replication (by activation of immune pathways or inhibition of crucial viral protein activity). To counter the host immune mechanism, the viral macrodomain removes ADP-ribose from the host or viral proteins, leading to an inactivation of host immune pathways or resumption of viral protein activity (Figure 4). Further in-depth studies are essential to identify and characterize other host targets and modes of action of macrodomain on modulating host/viral protein functions.

## Data Availability

Not applicable.

## Supplementary Materials

The following supporting information can be downloaded at: https://www.mdpi.com/article/10.3390/pathogens12050718/s1, Figure S1: Uncropped images of SDS-PAGE gel, ponceau stained and chemiluminescent substrate exposed membranes used in the study.; Figure S2: Sequence alignment of *Ae. aegypti* Tankyrase proteins and human PARP5b protein.; Figure S3 Sequence alignment of *Ae. aegypti* PARP protein and human PARP1 protein.; Figure S4: Sequence alignment of *Ae. aegypti* MARP protein and human PARP16 protein.; Figure S5: Coomassie brilliant blue stained SDS-PAGE gel and western blot of bacterial purified recombinant nsP3 protein.; Figure S6: Immunofluorescence assay of CHIKV infected Aag2 cells.
