# Supplementary material for: Characterization of an Aedes ADP-Ribosylation Protein Domain and Role of Post-Translational Modification during Chikungunya Virus Infection
Source: Pathogens. 2023 May 16;12(5):718. doi: 10.3390/pathogens12050718 (PMC10223269; doi:10.3390/pathogens12050718)
Supplement: Supplementary file 1 [file pathogens-12-00718-s001.zip › pathogens-2265931-supplementary.pdf]

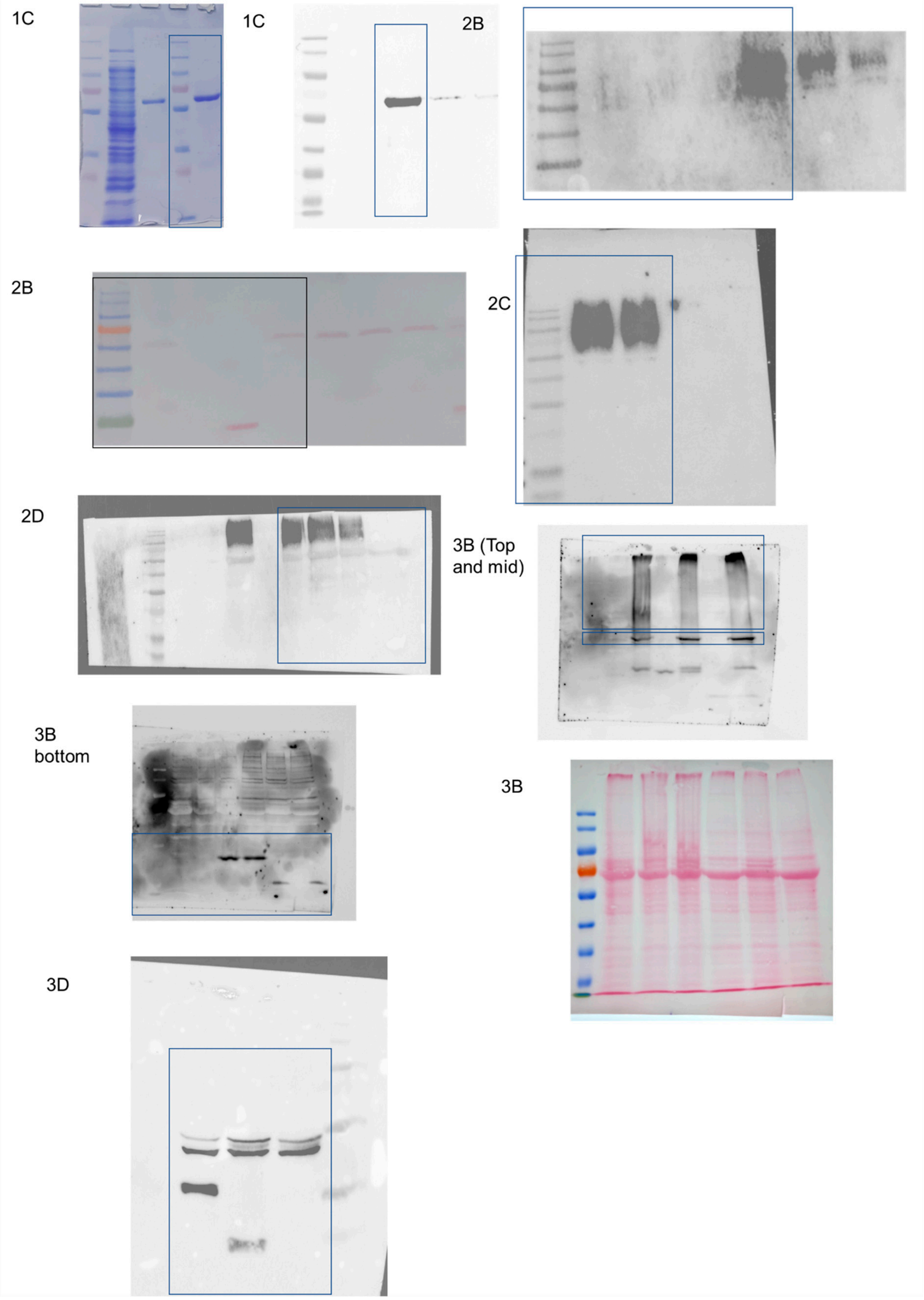

Figure S1 Uncropped images of SDS-PAGE gel, ponceau stained and chemiluminescent substrate exposed membranes used in the study. Number mentioned are figures where these are used the manuscript.

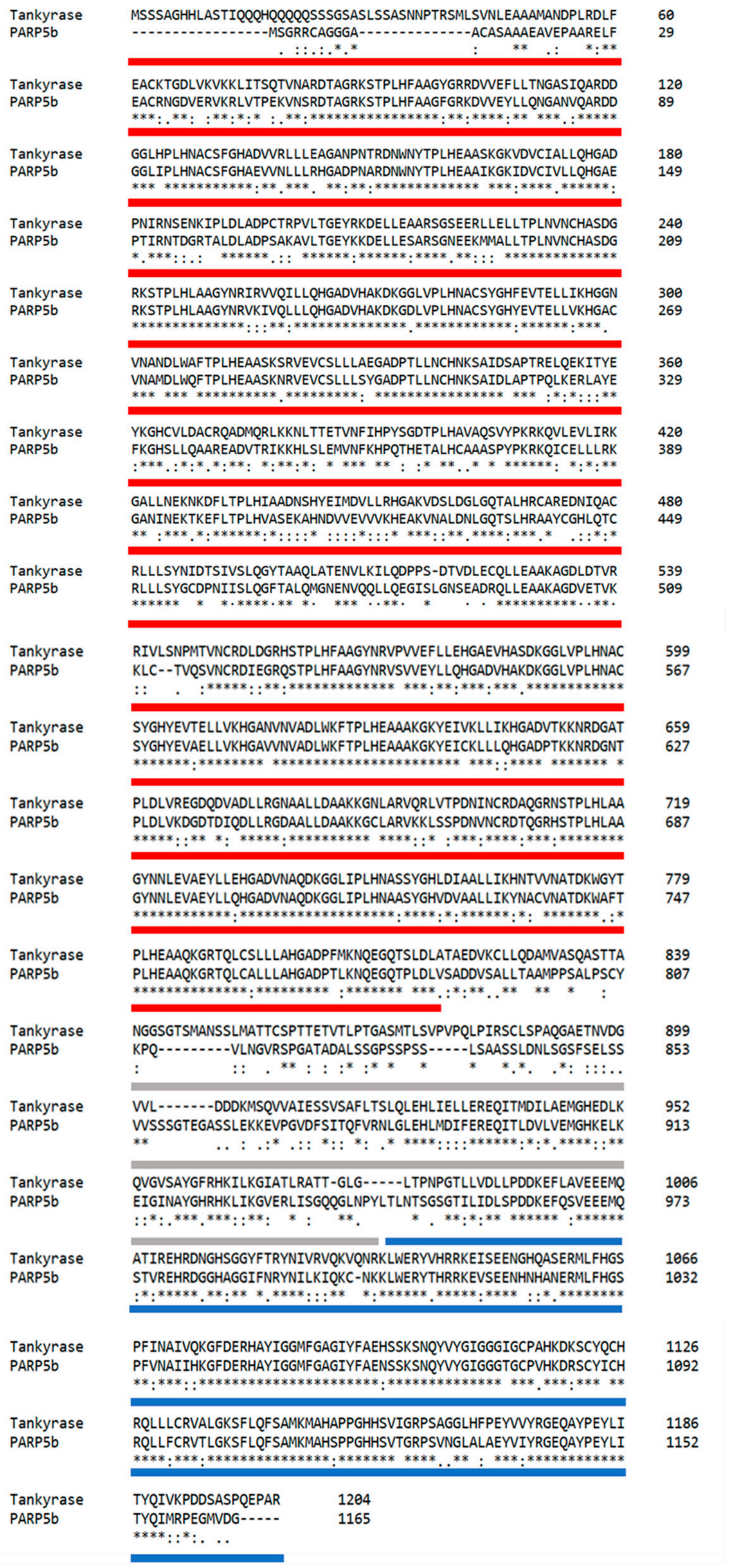

Figure S2 Sequence alignment of *Ae. aegypti* Tankyrase proteins and human PARP5b protein. The domains of *Ae. aegypti* Tankyrase were shown in coloured boxes. -Ankyrin repeats, -SAM domain, and -PARP catalytic domain. Asterisk (*) indicates fully conserved residue, colon (:) indicates conservation between groups of strongly similar properties, and period (.) indicates conservation between groups of weakly similar properties.

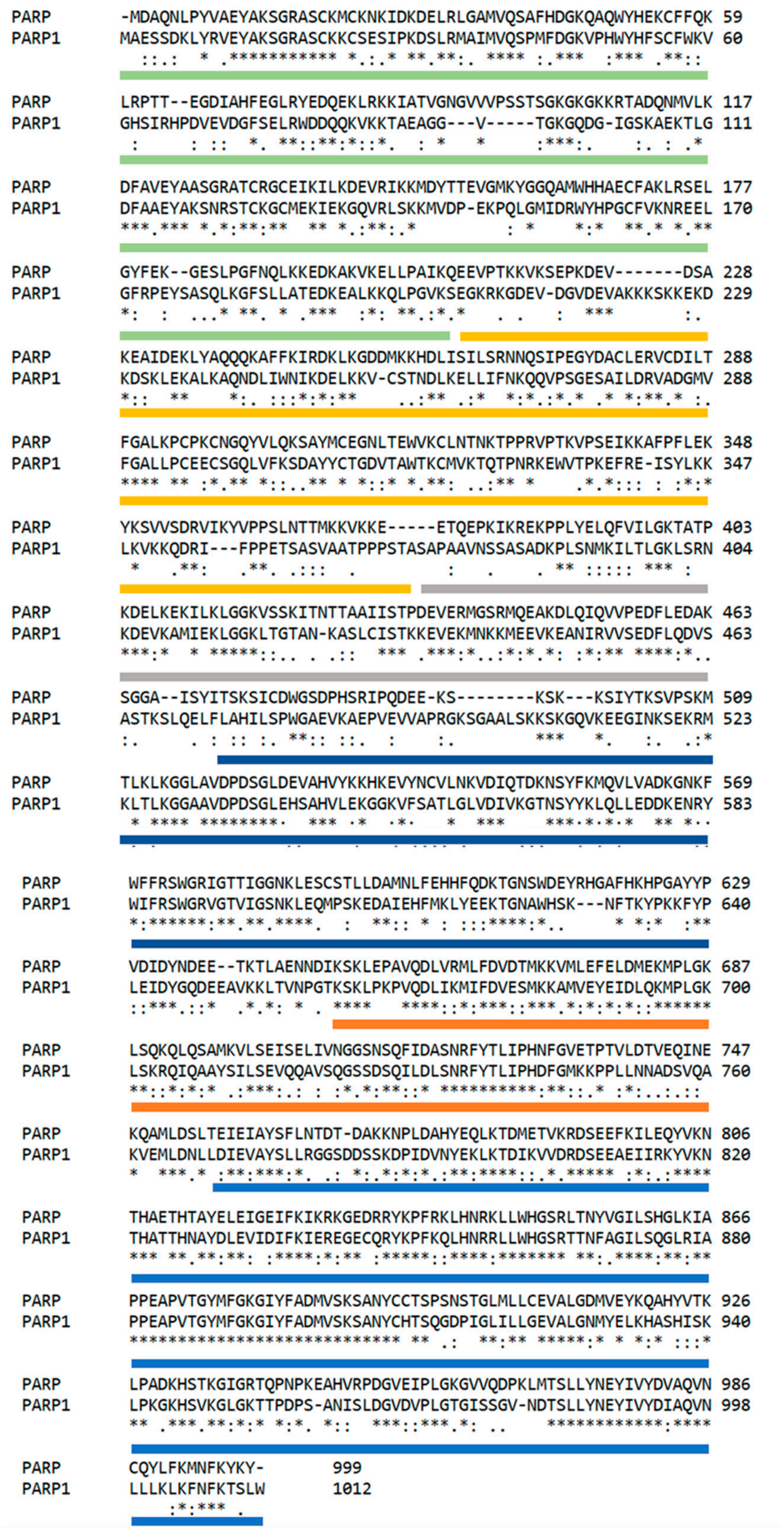

Figure S3 Sequence alignment of *Ae. aegypti* PARP protein and human PARP1 protein. The domains of *Ae. aegypti* PARP were shown in coloured boxes. ■-PARP Zinc finger, ■-PADR1, ■-BCRT domain, ■-WGR domain, ■-PARP alpha domain, and ■-PARP

catalytic domain. Asterisk (*) indicates fully conserved residue, colon (:) indicates conservation between groups of strongly similar properties, and period (.) indicates conservation between groups of weakly similar properties.

```
MARP      MESMYFSTQPDPSADSKRTILRELIDRDALAADLRLSLFVSAARSFKYDSCLQPFPPDFI    60
PARP16    ------------MQPSGWAAAREAAGRDMLAADLRCSLFASALQSYKRDSVLRPFPASYA    48
                      *  :  **  .** ****** ***.** :*:* ** *:*** .:

MARP      NGNKEKNIDELNRVLGGFQPLQEL--DIEDLNSKHLDLLHWILCQKSNPGLRTVPKKDFD    118
PARP16    RGDCKDFEALL-ADASKLPNLKELLQSSGDNHKRAWDLVSWILSSKVLT-IHSAGKAEFE    106
          .*: :.    *    . :  *:**  .  * :.:  **: ***..*    :::. * :*:

MARP      SILAK-APCIAAVQRPQQIFEVVYREDSNLERRFREKRDQFARQYAYHGSKLFNFYSILN    177
PARP16    KIQKLTGAPHTPVPAPDFLFEIEYFDPAN--AKFYETKGERDLIYAFHGSRLENFHSIIH    164
          .*    .   : *  *: :**: * : :*   :* *.:.:    **:***:* **:**::

MARP      YGLQQHLNRTALFGEGIYLSAELHVSQMFAPTGAGWARSALGSHLACTALCEYIDNPDYV    237
PARP16    NGLHCHLNKTSLFGEGTYLTSDLSLALIYSPHGHGWQHSLLGPILSCVAVCEVIDHPDV-    223
           **: ***:*:***** **:::* :: :::* * ** :* **  *:*.*:** **:**

MARP      KCHT---------------ENSIASDIPEKYILVKNNDLVQVRYLLVYGSNRRQVAPTEV    282
PARP16    KCQTKKKDSKEIDRRRARIKHSEGGDIPPKYFVVTNNQLLRVKYLLVYSQKPPKSR----    279
          **:*               ::* ..*** **::*.**:*::*:*****..:  :

MARP      SNNTPDQRMGQTTNGRAPPSPCVRWIAANKSWLMAGGYVLMLFGIGLMNNPNVHYMRQMF    342
PARP16    -----------------ASSQLSWFSSHWFTVMISLYLLLLLIVSVINSSAFQHF----    317
                           *  : *::::   :* . *:*:*: :.::*.  .:::

MARP      LQKINHVCHSLFGGVAERDA    362
PARP16    WNRAK------------R--    323
           :: :            *
```

Figure S4 Sequence alignment of *Ae. aegypti* MARP protein and human PARP16 protein. The domains of *Ae. aegypti* MARP were shown in coloured boxes. ▬-PARP catalytic domain. Asterisk (*) indicates fully conserved residue, colon (:) indicates conservation between groups of strongly similar properties, and period (.) indicates conservation between groups of weakly similar properties.

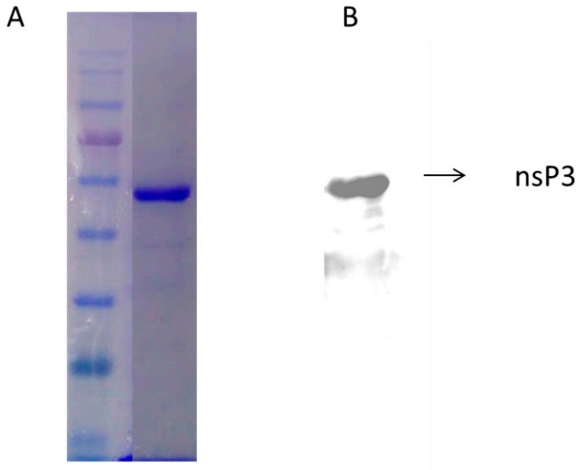

Figure S5 Coomassie brilliant blue stained SDS-PAGE gel and western blot of bacterial purified recombinant nsP3 protein.

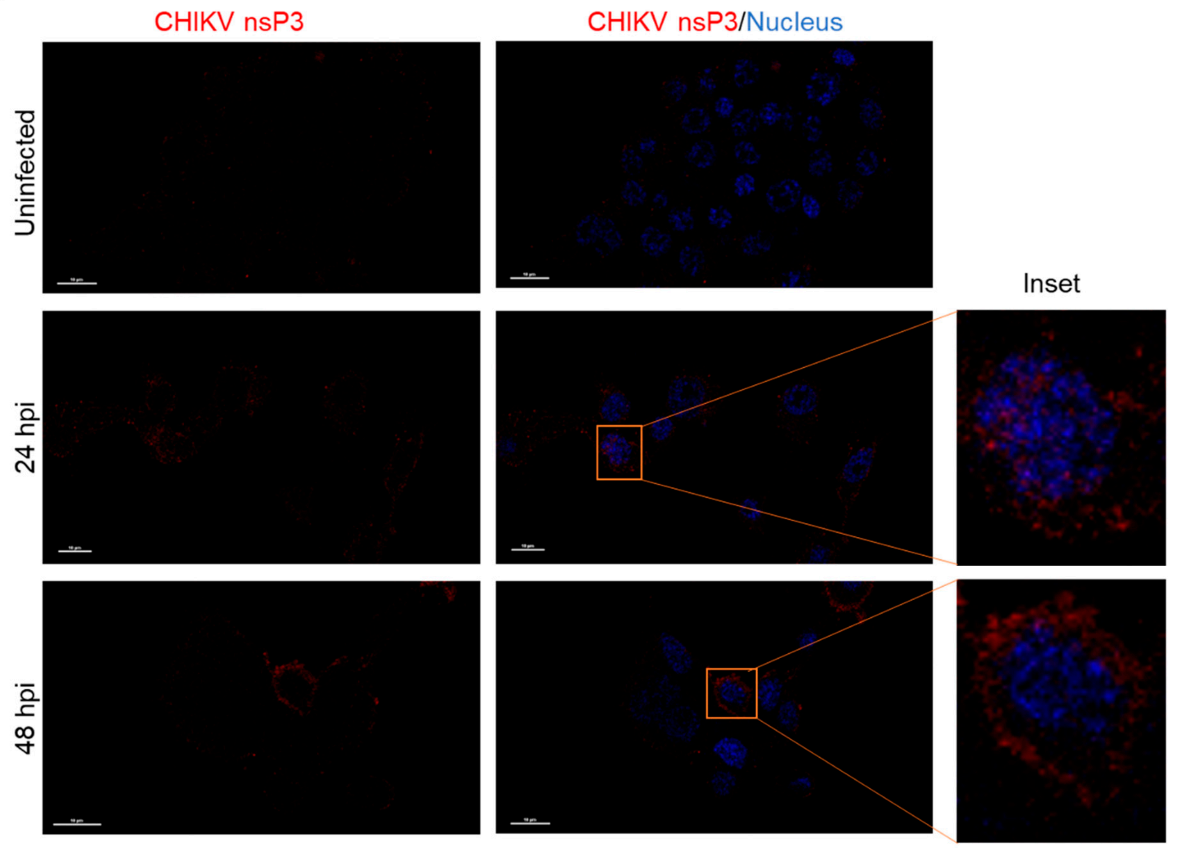

Figure S6 Immunofluorescence assay of CHIKV infected Aag2 cells. Aag2 cells were infected with CHIKV at MOI of 1 and fixed at 24 hpi and 48 hpi as well as uninfected cells. The cells were then incubated with anti-CHIKV nsP3 mice sera, followed by Alexa 594 anti-mice IgG antibody. The nucleus was stained with DAPI. Cells were visualized in confocal microscope at 100X with oil immersion. The insets represent magnification of selected zones. Bar length=10 μm.
